# Minor groove tetrads: a potent and versatile capping interaction for i-motif structures

**DOI:** 10.1007/s12551-026-01406-1

**Published:** 2026-01-22

**Authors:** Miguel Garavís, Bartomeu Mir, Israel Serrano-Chacón, Cristina Cabrero, Cristina Ugedo, Irene Gómez-Pinto, Núria Escaja, Carlos González

**Affiliations:** 1 Instituto de Química Física ‘Blas Cabrera’, CSIC, Serrano 119, 28006 Madrid, Spain; 2 Inorganic and Organic Chemistry Department, Organic Chemistry Section, and IBUB, University of Barcelona, Martí I Franquès 1-11, 08028 Barcelona, Spain; 3 BIOESTRAN Associated Unit UB-CSIC, Barcelona, Spain; 4 Present Address: Departament de Química, Universitat de les Illes Balears, Palma, Spain; 5 Present Address: Institute for Research in Biomedicine (IRB), Barcelona, Spain

**Keywords:** I-Motif, Tetrads, NMR, Non-canonical nucleic acids, Four-stranded structures

## Abstract

Minor groove tetrads (MGTs) have emerged as powerful structural elements capable of enhancing the stability and versatility of i-motif DNA structures. These non-canonical tetrads, formed by the minor groove association of two Watson–Crick or mismatched base pairs, act as capping platforms that reinforce hemiprotonated C:C⁺ stacks, enabling i-motif folding even at neutral pH. The resulting MGT-containing i-motifs (MGTiMs) display exceptional thermal and pH stability, tunable topology, and remarkable structural plasticity. Recent studies have revealed that MGTiMs can form compact architectures with only two C:C⁺ pairs, undergo reversible pH-dependent conformational transitions, and integrate seamlessly into duplex junctions without distorting B-DNA geometry. These insights may add new principles for rational i-motif engineering, guiding the design of predictable, homogeneous, and responsive DNA nanostructures. Furthermore, the synergy between MGT stabilization and chemical modifications, such as 2′-fluoro substitutions or fluorescent cytosine analogues, offers powerful tools for real-time structural monitoring and in-cell imaging. Beyond fundamental structural biology, MGTiMs hold strong potential for applications in biosensing, nanotechnology, and synthetic biology, providing programmable molecular systems that combine biocompatibility, robustness, and responsiveness to physiological stimuli.

## Introduction

DNA is far more than a passive carrier of genetic information. Over the past decades, it has become increasingly clear that nucleic acids can adopt a rich repertoire of non-canonical secondary structures that play active roles in the regulation of fundamental cellular processes. Among these, G-quadruplexes have been by far the most extensively studied (Huppert and Balasubramanian 2005; Todd et al. 2005; Chambers et al. 2015; Hänsel-Hertsch et al. 2016). Formed by guanine-rich sequences through the stacking of planar G-tetrads, G-quadruplexes have been detected genome-wide and implicated in transcription, replication, genome stability, and telomere maintenance. In contrast, their cytosine-rich counterparts, i-motifs, although structurally complementary and often encoded in the same genomic regions as G-quadruplexes, have historically received considerably less attention. This imbalance largely stems from the strong pH dependence of i-motif folding, which initially led to their classification as in vitro curiosities rather than biologically relevant DNA structures. However, accumulating biophysical, biochemical, and cellular evidence now demonstrates that i-motifs can form under near-physiological conditions and coexist dynamically with G-quadruplexes in vivo, suggesting that these two structures should be viewed not as isolated entities, but as interconnected components of a broader regulatory DNA structural landscape.

**Fig. 1:**
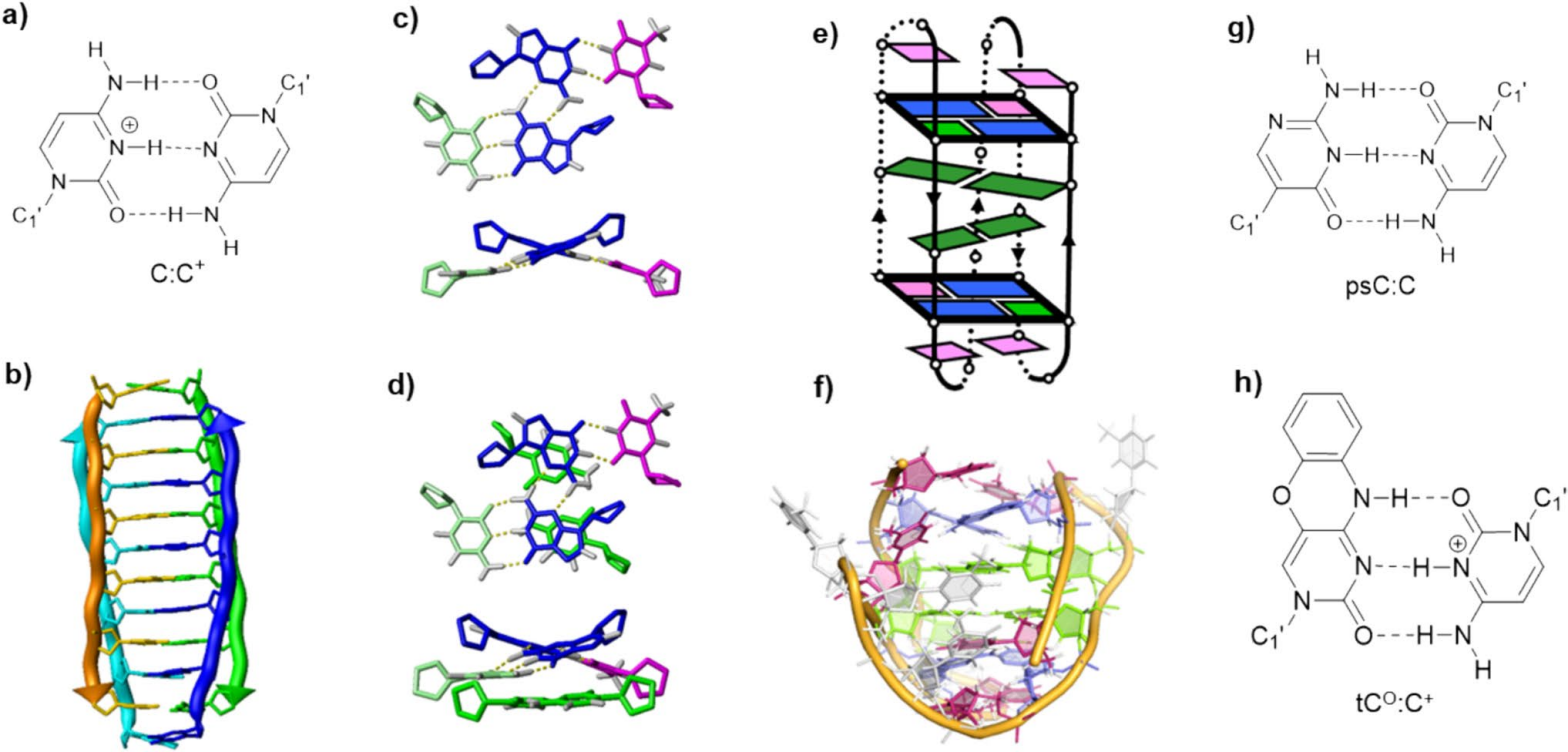
**a)** Hemiprotonated C:C⁺ base-pair; **b)** scheme of a tetramolecular i-motif (each strand in different colors); **c**) two views of the capping G:C:G:T MGT; **d)** detail of stabilizing interaction between the MGT and the adjacent C:C⁺ base pair; **e)** schematic view of a MGTiM; **f**) three-dimensional structure of the same MGTiM (PDB 5OGA); **g)** psC:C^+^ base pairs; **h)** tC^O^:C.^+^ base pair; figures of structural models are made with PyMol (Schrödinger and DeLano 2020)

The i-motif is a four-stranded DNA conformation composed of two parallel-stranded duplexes intercalated in antiparallel orientation and stabilized by hemiprotonated cytosine–cytosine base pairs (C:C⁺) (Fig. 1a, b) (Gehring et al. 1993). Its formation is strongly influenced by pH, because protonation of cytosine at position N3 is essential for the formation of the C:C⁺ base pair (Day et al. 2014; Abou Assi et al. 2018; Tao et al. 2024). Initially, i-motifs were regarded as exotic laboratory curiosities, assumed to form only under acidic conditions. However, live-cell imaging and immunochemical evidence have demonstrated i-motif formation inside cells (Zeraati et al. 2018; Cheng et al. 2021; Mir et al. 2024). The identification of naturally occurring i-motifs at near-neutral pH has increased the interest in their biological roles, which range from transcriptional regulation (Kaiser et al. 2017; Deep et al. 2025) to genome organization (Phan et al. 2000).

Beyond their emerging biological significance, i-motif structures have attracted considerable interest in nanoscience due to their reversible and finely tunable folding behavior. The strong dependence of i-motif stability on pH enables their use as molecular switches, pH-responsive nanodevices, and smart materials capable of converting chemical or environmental signals into mechanical or optical outputs. Such properties can be exploited in the design of nanosensors, drug delivery systems, and dynamic DNA nanostructures that respond to changing conditions. (Yatsunyk et al. 2014; Debnath et al. 2019; Pu et al. 2025; Luo et al. 2025).

Parallel to these discoveries, minor groove tetrads have emerged as a structural family of DNA motifs (Escaja et al. 2000, 2022). These tetrads are arrangements of four nucleobases, in which two Watson–Crick or mismatched base pairs associate through their minor groove sides creating non-planar four-base platforms distinct from the classical G-tetrads of guanine quadruplexes (Fig. 1c). Importantly, MGTs have been detected as integral capping elements in i-motifs structures, where they provide extraordinary stabilization of adjacent C:C⁺ pairs, dramatically increasing thermal and pH stability (Escaja et al. 2012; Mir et al. 2017a).

The discovery that MGTs can promote i-motif folding at neutral pH even in sequences with few cytosines has revealed new biological possibilities and engineering opportunities. This review synthesizes recent progress in the structural biology of MGT-containing i-motifs (MGTiMs)**,** drawing on recent key studies and highlights how these insights can be exploited for nanotechnology, biosensing, and aptamer design.

## Minor groove tetrads (MGTs)

Minor groove tetrads (MGTs) are a unique class of base-paired tetrads formed when two Watson–Crick or mismatched base pairs interact through their minor groove edges (Fig. 1c). This organization differs fundamentally from major groove tetrads, where base pairs associate via their major groove faces and remain nearly coplanar (Escaja et al. 2022). Major groove tetrads are planar and isomorphic to guanine tetrads and are most commonly found in the context of G-quadruplex structures (Kettani et al. 1995). In contrast, MGTs display a characteristic inclination of ~ 20–40° between the two interacting base pairs (Fig. 1c). This inclination impedes infinite stacking and renders MGTs incompatible with the planar arrangements typical of G-quadruplexes (Escaja et al. 2022). Another hallmark of MGTs is that their geometry brings phosphate backbones into unusually close proximity. While this could be electrostatically destabilizing, the effect is counterbalanced by favorable hydrophobic sugar–sugar contacts and, in some cases, by cation binding. Interestingly, this situation closely resembles the structural constraints found between adjacent antiparallel strands in i-motifs.

MGTs can adopt either direct or slipped arrangements, depending on the relative position of the paired bases (Escaja et al. 2007). In the direct configuration, the two base pairs align side by side and form bifurcated H(N2)(G)–O2(C) hydrogen bonds. In the slipped configuration, one base pair is shifted relative to the other, and stabilization arises from H(N2)(G)–N3(G) hydrogen bonds between neighboring guanines. Notably, in the context of i-motifs, only slipped MGTs have been observed so far.

A wide range of base combinations are known to form MGTs. G:C:G:C tetrads are the most stabilizing and have been identified in dimeric DNA loop structures such as d(GCATGCT) (Leonard et al. 1995), as well as in self-associating cyclic oligonucleotides, like d < pTGCTCGCT > and d < pCCGTCCGT > (Escaja et al. 2000, 2007). G:T:G:T tetrads, arising from two G:T mismatches, were initially described in dimeric assemblies and later recognized as the first examples of MGTs capping i-motif structures (Gallego et al. 1997; Escaja et al. 2012). G:C:G:T hybrid tetrads have been observed both in isolation (Viladoms et al. 2010; Mir et al. 2017a) and more frequently in association with i-motifs, where their stabilizing effect depends on sequence context. A:T:A:T and G:A:G:A tetrads are less common; although they have been detected in certain molecular environments, they have not yet been implicated in stabilizing i-motifs (Escaja et al. 2000; Kocman and Plavec 2017).

## The i-motif and the role of capping interactions

The i-motif is a four-stranded DNA structure formed by two parallel duplexes intercalated in opposite orientation. Its fundamental building block is the hemiprotonated C:C⁺ base pair, where two cytosines share a proton between their respective N3 positions, allowing the formation of an additional hydrogen bond (Benabou et al. 2014a, b; Abou Assi et al. 2018). In principle, this base pairing requires acidic or slightly acidic pH, which explains why i-motifs were long thought to occur only in in vitro acidic conditions and not relevant in biological processes. However, the local chemical environment can shift cytosine pK_a_, which can remain protonated at higher pH values. In fact, in recent years, a number of i-motif structures have been found in neutral conditions (Wright et al. 2017; Rogers et al. 2018; Školáková et al. 2023). In addition, *in cell* NMR, fluorescent probe studies and i-motif-specific antibodies have demonstrated i-motif formation inside living cells (Dzatko et al. 2018; Zanin et al. 2023; Víšková et al. 2024; Mir et al. 2024).

As in duplex DNA and G-quadruplexes, nucleotide sequence is a primary determinant of i-motif formation. Other key intrinsic factors that determine i-motif stability include cytosine tract length and loop length and composition (Fleming et al. 2017; Wright et al. 2017; Dzatko et al. 2018). Longer uninterrupted C-tracts generally favor folding, but specific sequence patterns can stabilize even very short tracts if supplemented by capping elements (Benabou et al. 2016; Cheng et al. 2021; Zhang et al. 2025). Loops connecting the intercalated duplexes can be short (named as class I, 2–4 nt) or long (class II, > 4 nt). Even i-motifs with 0 nucleotide loops have been recently reported (El-Khoury et al. 2023). Class II loops often provide additional stabilizing contacts, but systematic work shows that loop composition (e.g., thymine content) can be more critical than length alone (Fujii and Sugimoto 2015; Gurung et al. 2015; Reilly et al. 2015; Benabou et al. 2016; McKim et al. 2016).

Among all stabilizing elements, capping interactions at the termini of the C:C⁺ stack are crucial. Early studies highlighted the stabilizing effect of hairpin-like loops and base stacking, including non-canonical G:G pairs (Li et al. 2023; Guneri et al. 2024; Yu et al. 2012; Nesterova et al. 2015; Kaiser et al. 2017). Subsequent work has shown that minor groove tetrad capping can provide an additional and highly effective stabilization mechanism (Escaja et al. 2012; Serrano-Chacón et al. 2023).

## i-Motif and minor groove tetrads: the MGTiM concept

The discovery that MGTs can flank and stabilize i-motif cores has given rise to a distinct structural family: the MGT-containing i-motifs (MGTiMs). In these DNA structures, MGTs typically flank the terminal C:C⁺ base pairs, forming a stabilizing “cap” that protects the C:C⁺ stack from solvent and increases the effective pH at which the i-motif remains folded (Fig. 1d-–f) (Mir et al. 2017a). This capping can raise the pH midpoint (pH₁_/_₂) of folding well above neutrality and can boost melting temperatures (Tₘ) by 10 °C or more compared to uncapped analogues. In other words, MGTs have an extraordinary capacity for enhancing thermal and pH i-motif stability.

Several well-characterized sequences exemplify this architecture. Early NMR studies of short repetitive oligonucleotides, such as d(TCGTTTCGT), demonstrated that they fold into compact dimeric i-motifs stabilized by two G:T:G:T MGTs (Escaja et al. 2012). Later studies on related sequences d(YCGXXYCG) (where Y = C/T and X = any nucleotide) showed that G:C:G:C and G:C:G:T MGTs are even more stabilizing (Serrano-Chacón, et al. 2023). Moreover, two repeats of these sequences connected by loops of different size, ranging from 2 to 7 nucleotides, fold into analogous monomeric i-motif structures (Mir et al. 2017a). These so-called minimal i-motifs require only two C:C⁺ base pairs, in sharp contrast to classical i-motifs, which typically contain four or more. Notably, sequences composed of two TCGTTCCGT or CCGTTCCGT repeats exhibit melting temperatures up to 47 °C at pH 5 and approximately 30 °C at pH 7, despite containing only two C:C⁺ pairs.

A systematic analysis of the loop regions in these motifs shows that while the G–XX–Y loop must comprise one or two nucleotides (Escaja et al. 2013), the linker loop between the two repeats can vary widely in length without disrupting the overall structure. Bioinformatic analyses indicate that sequences matching the consensus d(YCGXXYCG–Xₙ–YCGXXYCG) are abundant in the human genome and enriched in regulatory regions such as promoters and enhancers. Moreover, these repeats can form tandem arrays, giving rise to higher-order “bead-on-a-string–like” MGTiM superstructures (Mir et al. 2017a).

The ability of MGTiMs to fold at, or near, neutral pH suggests that they are not merely in vitro curiosities. Their enrichment in promoter regions, combined with strong evidence of i-motif formation in living cells (Mir et al. 2024), points to potential roles in gene regulation and genome architecture.

## MGTs and C:C⁺–induced stability

Classical i-motifs are stabilized by four or more hemiprotonated C:C⁺ base pairs, but systematic studies on MGT-capped systems demonstrate that two C:C⁺ base pairs flanked by two MGTs can rival or exceed the stability of longer C-tract i-motifs (Wright et al. 2017). The main distinctive stabilizing factor is the favorable interaction between the two guanines in the MGT with adjacent positively charged C:C⁺ pairs (Fig. 1d). QM theoretical calculations clearly show that this pi/cation interaction shift the pK_a_ of the hemiprotonated C:C^+^ base pair by almost 4 pH units, in notable agreement with experimental observations (Serrano-Chacón et al. 2023).

Although this interaction is common to all described MGTs, the sequence context strongly influences which MGT forms. For example, recent systematic analysis revealed that, at neutral pH, G:C:G:C and G:C:G:T tetrads confer the greatest stabilization, whereas G:T:G:T tetrads provide a weaker effect (see Table 1) (Serrano-Chacón et al. 2023; Ashida et al. 2025). Within G:C:G:T tetrads, the orientation of G:C vs. G:T relative to the adjacent C:C⁺ pair significantly affects thermal stability. Other position-specific effects, such as a preference for thymine at the 3’ end of the long loop, likely arise from subtle steric and hydration factors in the loop and groove regions, underscoring the finely tuned interplay between MGT identity, positioning, and i-motif geometry (Ashida et al. 2025; Mir 2026).

**Table 1:** Reported i-motif–forming sequences containing MGTs as capping elements, including the corresponding reference, type of MGTs in each case, PDB code (when available), and melting temperature (Tₘ) at the indicated pH. Chemically modified residues are shown in bold (**psC**, pseudoisocytidine; **fC**, 2′-fluoroarabinocytidine; **tC**^**O**^, phenoxazine derivative)

Reference	Sequence	MGT	PDB code	Tm (°C)/pH
(Gallego et al. 1997)	d(TCCCGTTTCCA)	1×G:T:G:T	1C11	~ 50/4.1
(Escaja et al. 2012)	d<pTCGTTTCGTT>	2×G:T:G:T	2LSX	42.0/4.5
(Mir et al. 2017b)	d(TCCGTTTC**psC**GT)	2×G:G	5NIP	16.7/7.0
(Mir et al. 2017a)	d(TCGTTCCGT-T_3_- TCGTTCCGT)	2×G:C:G:T	5OGA	32.1/7.0
(Mir et al. 2017a)	d(TCGTTTCGT-T_4_- TCGTTTCGT)	2×G:T:G:T	-	16.6/7.0
(Serrano-Chacón et al. 2021)	d(CCCGTTTCCT-CGCGAAGCATTCGCG-CCCGTTTCCT)	1×G:T:G:T	7O5E	26.9/7.0
(Serrano-Chacón et al. 2023)	d(CCGTTCCGT-T_4_- CCGTTCCGT)	2×G:C:G:C	8BQY	27.8/7.0
		2×G:T:G:T	8BV6	
(Cabrero 2023)	d(CCGTT**fC**CGT-T_4_- CCGTTCCGT)	2×G:C:G:C	-	27.6/7.0
		2×G:T:G:T		
(Cabrero 2023)	d(**fCfC**CGTTC**fCfC**C-GGAAGCATTCC-**fCfC**CGTTC**fC**C)	1×G:C:G:C	-	48.2/7.0
(Mir et al. 2024)	d(C**tC**^**O**^GTTCCGT-T_4_- CCGTTCCGT)	2×G:C:G:C	8OFC	38.8/7.0
(Tsvetkov et al. 2025)	d(CGCTCACG-CCCCGTTTCCCCCCC CCCCGTTTCCCCC-CGTGAGCG)	1×G:T:G:T	8S4N	n.d
(Ashida et al. 2025)	d(TCGTTCCGT-A_3_-TCGTTCCGT)	2×G:C:G:T	-	36.0/6.0
(Ashida et al. 2025)	d(CCGTTCCGT-A_3_-TCGTTCCGT)	1×G:C:G:C 1×G:C:G:T	-	37.0/6.0
(Ashida et al. 2025)	d(TCGTTCCGT-A_3_-CCGTTCCGT)	1×G:C:G:C 1×G:C:G:T	-	35.0/6.0
(Ashida et al. 2025)	d(TCGTTTCGT-A_3_-TCGTTCCGT)	1×G:T:G:T 1×G:C:G:T	-	31.7/6.0
(Ashida et al. 2025)	d(TCGTTCCGT-A_3_-TCGTTTCGT)	1×G:T:G:T 1×G:C:G:T	-	30.7/6.0

## MGT-dependent conformational switches

A unique feature of some MGTiMs is their ability to reorganize capping tetrads in response to pH or other environmental stimuli without losing their overall i-motif fold. The ability of supporting reversible pH-driven transitions underpins the potential use of MGTiMs as reversible molecular switches.

**Fig. 2:**
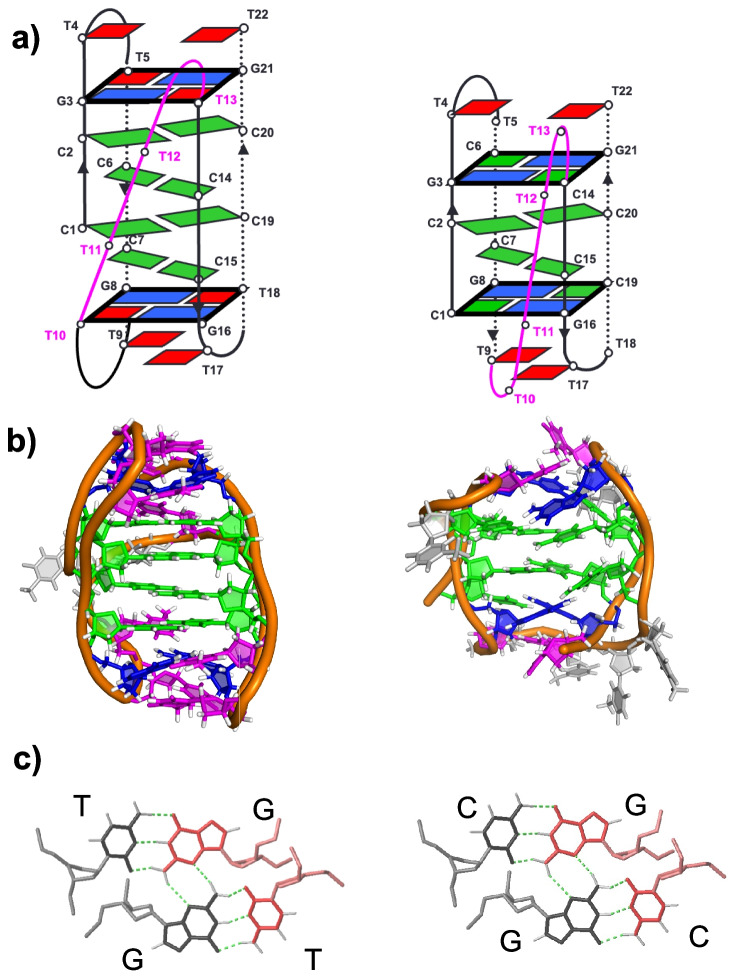
Two structures of the same i-motif forming sequence. Left: Structure at acidic pH (PDB 8BQY). Right: Structure ant neutral pH (PDB 8BV6). Schematic view (**a**), three-dimensional structure (**b**), and minor groove tetrads involved in each structure (**c**). Structural models in panel b) were made using PyMol (Schrödinger and DeLano 2020)

I-motifs stabilized by capping tetrads containing G:C base pairs are unusual because they contain both neutral and protonated cytosines under the same experimental conditions. The simultaneous presence of C:C⁺ and G:C base pairs raises questions about the impact of pH on the stability of these structures, since the stabilizing effect of G:C-containing tetrads might be compromised under acidic conditions. In-depth structural analyses of MGTiMs containing G:C:G:C tetrads have shown that these i-motifs can undergo dramatic yet reversible conformational changes triggered by pH variation (Serrano-Chacón et al. 2023). NMR and circular dichroism studies revealed that different MGT combinations stabilize distinct conformers. For example, sequences with two CCGTTCCGT repeats can shift from a G:C:G:C-capped neutral-pH conformation to a G:T:G:T-capped acidic-pH form (Fig. 2). In this transition, the cytosines, that at neutral pH participate in the Watson–Crick G:C base pairs of the MGTs, form C:C⁺ base pairs in the central part of the i-motif under acidic conditions, producing a more elongated structure. This reversible transition does not require complete unfolding; rather, the capping tetrads reorganize while part of the intercalated C:C⁺ core remains intact and additional C:C⁺ base pairs are formed. The process depends entirely on the protonation state of specific key cytosines (Serrano-Chacón, et al. 2023).

This illustrates the structural plasticity of MGTiMs and their potential as dynamic pH sensors. This unprecedented conformational transition widens the range of pH-dependent changes associated with i-motifs and can lead to a new type for pH sensors. Furthermore, it opens the range of potential i-motif structures that can be stable under physiological conditions.

## Engineering i-motifs through incorporating MGTs

The strong stabilization conferred by MGTs offers valuable design principles for engineering i-motifs with tailored stability, topology, and responsiveness. A key strategy for stabilizing i-motifs is to enforce proper alignment of cytosine tracts, ensuring that C:C⁺ base pairs intercalate in a defined register. Rational sequence design enables the minimization of competing alternative folds, thereby producing homogeneous and well-defined i-motif species, an especially desirable feature for solution NMR studies. This approach is particularly effective when combined with capping interactions such as MGTs, which provide additional stabilization and allow fine-tuning of i-motif conformational behavior.

**Fig. 3:**
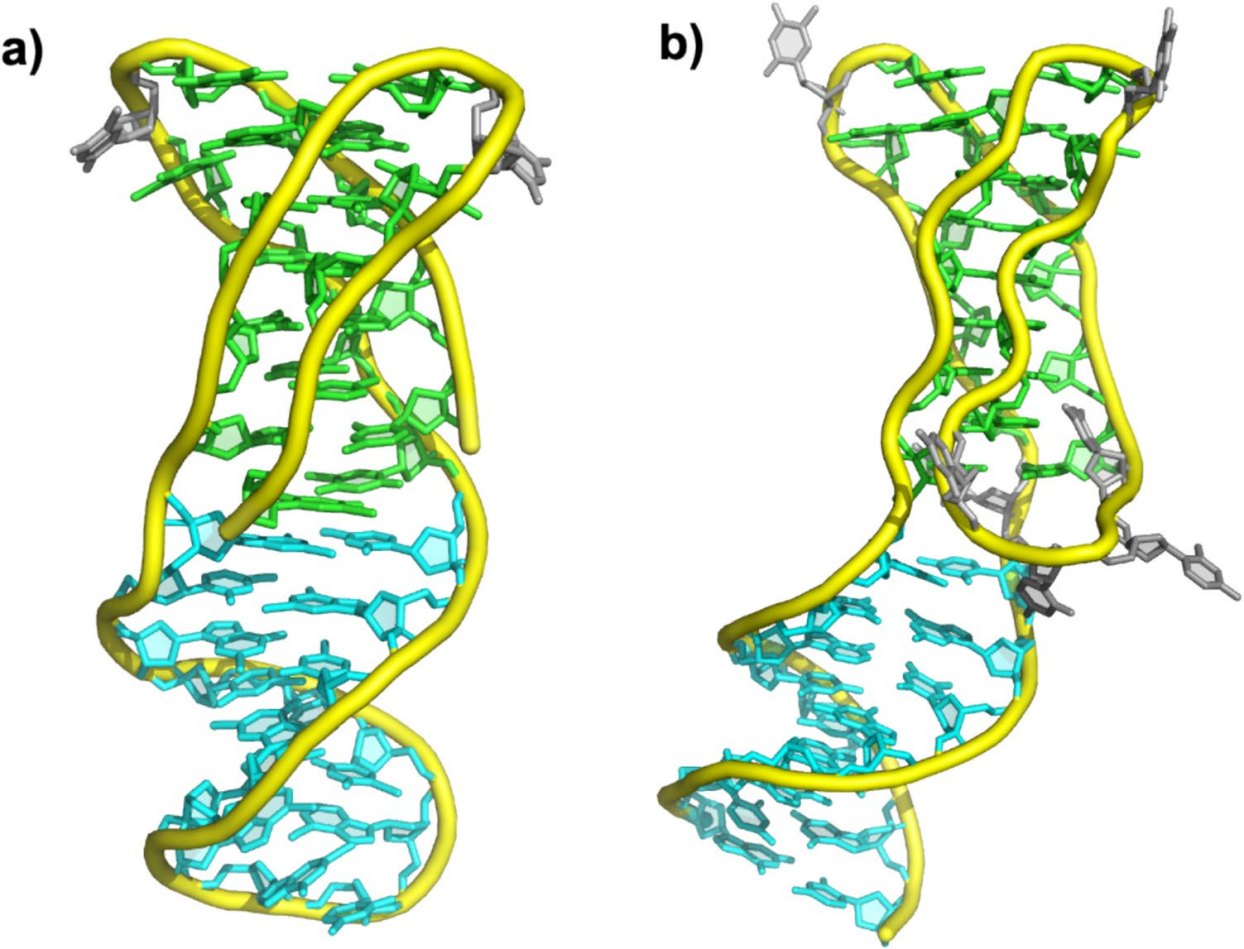
Examples of i-motif/duplex junctions. **a**) Structure of an IDJ with a rigid interface between the i-motif and B-DNA moieties (PDB 7O5E); **b)** Structure of the IDJ found in aptamer where the interface is flexible (PDB 8S4N). Color code: i-motif resides in green, B-DNA resides in cyan, flexible residues in gray. Figure made with PyMol (Schrödinger and DeLano 2020)

A central application of this design principle is the construction of i-motif/duplex junctions (IDJs), in which an i-motif domain is seamlessly connected to a canonical duplex segment. Solid evidence for i-motif formation in cells has accumulated in recent years (Dzatko et al. 2018; Zeraati et al. 2018; Zanin et al. 2023; Víšková et al. 2024). Because this formation is transient and localized, whereas most genomic DNA remains in the B-form, junctions between these two types of DNA structures must naturally occur in vivo. Recent structural studies have shown that such hybrid constructs are stable at neutral pH when flanked by appropriate capping tetrads (e.g., G:T:G:T). The first high-resolution structure of an IDJ demonstrated that i-motif and B-DNA regions can coexist without significant distortion of base stacking (Serrano-Chacón et al. 2021). The interface is mediated by a combination of C:C⁺ stacking continuity and stabilizing mismatches (e.g., T:T), which preserve the integrity of the junction while enabling a smooth structural transition between i- and B-DNA domains (Fig. 3a).

This work highlights how careful sequence placement of cytosines and thymines can direct the formation of MGTs on one side of the i-motif, while the opposite side is linked to duplex DNA. The resulting constructs display dual groove morphologies and retain robust folding across a broad pH range. Importantly, variations at the interfacial base-pairs (e.g., replacing T:T with C:C⁺) significantly modulate the thermal stability and flexibility of the junction. Such modifications can pre-program the topology of the i-motif, favoring a specific register and reducing polymorphism.

IDJs therefore represent a generalizable design for enforcing C:C⁺ register while exploring new functional architectures with multiple applications. By combining the structural versatility of i-motifs with the recognition properties of duplex DNA, these engineered junctions create opportunities for constructing biosensors and nanodevices, in which the i-motif domain can function as an environmentally sensitive structural switch, while the duplex region offers a programmable scaffold for sequence-specific recognition or probe immobilization (Nesterova and Nesterov 2014; Hu and Ying 2023; Luo et al. 2025). Such architectures are well suited for molecular beacons, aptamer-based sensors, and nanodevices in which binding of a target molecule or a local pH change induces a detectable conformational or optical response, for example, through fluorescence activation, FRET efficiency changes, or altered binding affinities (Modi et al. 2013; Dembska et al. 2017; He et al. 2021; Alieva et al. 2025).

As occurs with G-quadruplex/duplex junctions (Chu et al. 2019; Ngoc et al. 2020; Kotkowiak and Pasternak 2021), IDJs offer a powerful framework for selective molecular recognition. The junction region between i-DNA and B-DNA generates unique three-dimensional features, including peculiar groove shapes, mixed electrostatic environments, and discontinuities in base stacking that are absent in either structure alone. These structural signatures create well-defined binding pockets that can be selectively recognized by small molecules, peptides, or proteins, enabling discrimination between closely related DNA conformations. Such selectivity is particularly relevant for the development of ligands that target regulatory DNA elements without broadly interacting with canonical duplex DNA (Rodriguez et al. 2023).

Moreover, IDJs serve as valuable structural mimics of biologically relevant DNA motifs found in gene promoters, such as those of the *KRAS* and *NMYC* oncogenes, where i-motif formation is thought to coexist with duplex regions and to modulate transcriptional activity (Benabou et al. 2014a, b; Kaiser et al. 2017). By reproducing these hybrid architectures in a controlled and stable manner, engineered IDJs may provide tractable model systems for studying protein–DNA recognition, screening selective ligands, and designing functional nanodevices. Altogether, the principle of enforcing C:C⁺ register through rational sequence design emerges as a strategy for generating stable and predictable i-motif–based systems.

Finally, recent studies have demonstrated that IDJ-containing aptamers can achieve exceptional binding specificity and affinity. For example, an influenza A virus aptamer incorporating both i-motif and B-DNA elements exhibited enhanced structural stability and improved target recognition. Further sequence optimization, through the introduction of a minor groove tetrad (MGT) on one side of the i-motif domain, enabled the design of a shorter and more stable construct that could be thoroughly characterized by NMR and other biophysical techniques (Tsvetkov et al. 2025). In contrast to previously determined IDJ structures, in this case, the i-motif and duplex regions are not in direct contact but are separated by a short, flexible linker, providing additional conformational adaptability (Fig. 3b).

## MGTs and chemical modifications

Epigenetic base modifications, particularly cytosine methylation, add a layer of complexity to i-motif stability and regulation. In canonical i-motifs, 5-methylcytosine (5mC) has been shown to exert context-dependent effects, influencing both the pKₐ of cytosine protonation and base stacking interactions, and thereby modulating folding equilibria (Školáková et al. 2020; Deep et al. 2025). In the case of MGTiMs, epigenetic modifications are expected to have a more pronounced structural impact due to the tight stacking interactions between the capping tetrads, involving neutral cytosines, and the hemiprotonated C:C⁺ core. Preliminary and as yet unpublished results suggest that methylation of cytosines participating in the minor groove tetrad tends to exert a stabilizing effect. In contrast, methylation of cytosines involved in C:C⁺ base pairs produce a more complex and sequence-dependent response, potentially reflecting competing effects on protonation equilibria, intercalation geometry, and local electrostatics. These observations point to epigenetic modification as a powerful, but still underexplored, modulator of MGTiM structure, and stability (unpublished results).

Other non-natural chemical modifications provide an additional level of control over i-motif stability and responsiveness. In the context of MGT-containing i-motifs, the synergy between chemical analogues and capping tetrads is particularly powerful. Incorporation of pseudoisocytidine (psC), a neutral analogue of protonated cytidine (Fig. 1g), into MGTiMs shows that neutral psC:C base pairs can stabilize i-motifs at neutral pH only when the psC:C pairs are located at the ends of the intercalated C:C⁺ stacks. Remarkably, in this case, only G:G mismatches are identified as capping elements of the structure. Otherwise, the resulting i-motifs are observed only at acidic pH and not stabilized by neutral psC residues. The formation of psC:C⁺ or psC:psC⁺ hemiprotonated base pairs appears to be required, indicating that positively charged base pairs are essential for stabilizing this non-canonical DNA structure (Mir et al. 2017b).

Recent advances have highlighted the use of fluorescent cytosine analogues such as 1,3-diaza-2-oxophenoxazine (tC^O^) (Fig. 1h), which not only participates in canonical and hemiprotonated base-pairing but also offers unique photophysical properties (Mir et al. 2024). When incorporated adjacent to a G:C:G:C minor groove tetrad, tC^O^ forms a hemiprotonated tC^O^:C⁺ pair that stacks efficiently with the tetrad guanines. This interaction induces remarkable stabilization, raising melting temperatures by nearly 10 °C at neutral pH. NMR and quantum mechanical calculations demonstrate that the aromatic surface of tC^O^ enhances π-stacking with the tetrad, providing ~ 3 kcal/mol additional stabilization compared to natural cytosine. Consequently, tC^O^-containing MGTiMs can remain folded at physiological pH values where unmodified analogues are mostly unfolded.

The fluorescence of tC^O^ is highly sensitive to its microenvironment. In duplex DNA, Watson–Crick G:tC^O^ pairs yield bright emission with minimal quenching. By contrast, in MGTiMs, the formation of hemiprotonated tC^O^:C⁺ base pairs adjacent to tetrads provokes strong fluorescence quenching at neutral pH. This property enables direct, real-time monitoring of folding and unfolding transitions, both in vitro and inside cells. Experiments with HeLa cells have confirmed that tC^O^-labeled MGTiMs undergo pH-driven folding transitions that can be tracked optically, establishing tC^O^ as a minimally perturbing and highly informative probe (Mir et al. 2024).

The comparison of tC^O^ incorporation at different positions shows that stabilization is context-dependent. For instance, when tC^O^ replaces a cytosine involved in a C:C⁺ pair next to a tetrad, exhibits significantly greater stabilization and fluorescence quenching than when tC^O^ is positioned within a G:C base pair of the tetrad. These findings highlight that the interplay between tetrad identity, local protonation state, and the presence of aromatic analogues can finely tune i-motif thermodynamics and optical responses.

Beyond fluorescent analogues, other chemical modifications compatible with MGTiMs include 2′-fluoro-arabino substitutions, which are highly efficient i-motif stabilizers (Assi et al. 2016). When combined with tetrad stabilization, these modifications enable the design of highly stable, biocompatible, and responsive nucleic acid devices. Moreover, ^19^F NMR spectroscopy in these systems provides a particularly powerful tool for monitoring slow conformational transitions and for distinguishing folded, intermediate, and unfolded species (Cabrero 2023).

Altogether, MGTiMs provide a unique platform where chemical modifications not only reinforce structural stability but also introduce powerful functional readouts. The case of tC^O^ demonstrates that MGT–analog synergy can transform i-motifs into robust fluorescent sensors and in-cell probes, paving the way for applications in nanotechnology, diagnostics, and synthetic biology.

## Future directions and potential applications

The rapid progress in understanding and exploiting MGTiMs has opened several promising avenues for future research. While i-motif formation has been visualized in living cells, comprehensive genome-wide mapping of MGTiM loci remains lacking. High-throughput chemical mapping and single-molecule imaging could identify their genomic distribution and elucidate potential roles in transcriptional regulation, replication, and genome organization. The development of new fluorescent probes, isotope-labeled oligonucleotides, and fluorine-containing derivatives will further enable real-time monitoring of MGTiM folding and dynamics in living cells.

In addition to these imaging and detection tools, it will be important to assess whether the existing i-motif–specific antibody, iMab, can also recognize MGTiMs, or whether the presence of capping MGTs hinders antibody binding. Developing new, highly selective antibodies or ligands capable of specifically detecting MGTiMs would greatly enhance our ability to visualize these structures within cells and monitor their dynamics across different stages of the cell cycle.

Understanding how proteins interact with IDJs represents another promising direction for future research. IDJs located near promoter regions can serve as dynamic structural elements that influence transcriptional regulation. A notable example is the heterogeneous nuclear ribonucleoprotein K (hnRNP K), a multifunctional protein known to bind C-rich DNA and RNA sequences. The interaction of hnRNP K with DNA regions capable of forming i-motif/duplex junctions has been shown to enhance KRAS oncogene transcription, thereby linking the structural dynamics of IDJs to gene expression control (Kaiser et al. 2017). The ability of MGTs to stabilize these junctions under near-physiological conditions makes these constructs an especially attractive model system for investigating sequence- and conformation-specific protein recognition.

Moreover, MGTiMs provide excellent scaffolds for the rational design of responsive DNA nanodevices. Next-generation DNA nanomachines may function as multi-input logic gates, integrating pH with other stimuli such as ions or redox state, or as precision drug-delivery systems that release their cargo under specific intracellular conditions. The combination of structural robustness, pH responsiveness, and sequence programmability makes MGTiMs particularly attractive for these applications. I-motifs have long been recognized as pH-responsive elements with interesting applications as pH sensor. MGT-capped systems push this concept further by allowing precise control over operational pH windows much closer to physiological pHs.

Since intracellular pH is a hallmark of many pathological states (e.g., acidification during apoptosis or cancer progression), MGTiMs can be integrated into diagnostic probes and drug-delivery systems that activate in response to disease-specific pH environments. In this sense, the fluorescent cytosine analogue tC^O^ has proven particularly useful for in vivo imaging of i-motif folding and for monitoring pH in live cells.

The exceptional thermal stability of MGTiMs enables their incorporation into DNA nanostructures as structural reinforcements or dynamic hinges. Their ability to fold into tandem “rosary bead-like” superstructures provides additional opportunities for constructing long, periodic nano-architectures. MGTiMs are also suitable building blocks for synthetic gene regulatory networks and bioelectronic interfaces. Their predictable behavior can be harnessed to create molecular devices that respond to cellular signals and modulate gene expression or metabolic pathways. Moreover, engineering hybrid DNA constructs that combine MGTiMs with other non-canonical motifs, such as G-quadruplexes or triplexes, hold great promise for developing complex and programmable nanodevices.

Finally, the exceptional chemical and thermal robustness of MGTiMs makes them attractive candidates for synthetic biology applications. Incorporating MGTiMs into synthetic gene networks could enable tunable gene-expression modules responsive to metabolic or microenvironmental cues. Potential therapeutic applications include smart antivirals, cancer diagnostics, and controlled gene editing, leveraging the predictable folding and dynamic behavior of MGTiMs.

## Concluding remarks

Minor groove tetrads have transformed our understanding of i-motif stability and expanded the design space for DNA structures. Through a combination of innovative sequence engineering, chemical modification, and structural analysis**,** we are beginning to harness MGTiMs not only as fundamental models of DNA structural plasticity but also as potential platforms for pH-responsive devices and therapeutics**.** Continued integration of experimental and computational approaches promises to unlock the full potential of these remarkable nucleic acid architectures.

## Data Availability

No datasets were generated or analyzed during the current study.
